# Long-Term Clinical and Safety Outcomes of Canaloplasty Performed across All Grades of Glaucoma Severity

**DOI:** 10.1155/2023/5625990

**Published:** 2023-05-10

**Authors:** Shamil Patel, George Reiss

**Affiliations:** Banner-University Medical Center Phoenix, Phoenix, USA

## Abstract

**Purpose:**

To investigate the clinical effectiveness of canaloplasty performed with an ab interno technique using the iTrack microcatheter (Nova Eye Medical) in patients with mild-moderate glaucoma as compared to severe glaucoma.

**Methods:**

This is a retrospective single-center case series. Patients were preoperatively categorized as mild/moderate vs. severe glaucoma assessed using the mean deviation (MD) score and controlled group (baseline intraocular pressure (IOP) ≤18 mmHg) vs. uncontrolled group (>18 mmHg). All patients with glaucoma were eligible for recruitment except those who had undergone previous glaucoma surgery (with the exception of selective laser trabeculoplasty or SLT). Patients underwent canaloplasty via an ab interno surgical technique with or without phacoemulsification and were monitored for IOP, glaucoma medication usage, and surgical complications.

**Results:**

In total, 72 eyes were followed for 3.4 ± 0.5 years. Mean pre-op IOP (mmHg) was 19.3 ± 7.7 in the standalone group (*n* = 9) and 18.5 ± 5.6 in the combined group (*n* = 63) (*p*=0.38). At the last follow-up, mean IOP reduced by 36% to 12.4 ± 4.4 (*p*=0.02) in the standalone group and by 26% to 13.7 ± 4.8 in the combined group (*p* < 0.001). Mean pre-op IOP (mmHg) was 18.6 ± 5.2 in the severe group (*n* = 24) and 18.6 ± 6.2 in the mild-moderate group (*n* = 48) (*p*=0.48). Mean IOP was 14.1 ± 6.3 (−24%; *p* < 0.001) and 13.3 ± 3.7 (−29%; *p* < 0.001), respectively, at the last follow-up. Mean glaucoma medication usage decreased from 2.5 ± 0.9 to 2.1 ± 0.9 (−15%; *p*=0.083) in the severe group and 2.3 ± 1.0 to 1.4 ± 1.3 (−40%; *p* < 0.001) in the mild/moderate group. There was one localized Descemet's membrane detachment in the moderate group.

**Conclusion:**

iTrack canaloplasty achieved a statistically significant IOP reduction in mild-moderate and severe eyes and was found to be an effective option for reducing IOP and medications in patients with mild-moderate primary open-angle glaucoma (POAG). In severe eyes, it has reduced IOP while the medications remained stable.

## 1. Introduction

Glaucoma is a sight-threatening optic neuropathy that can cause irreversible loss of vision. It is the second most common cause of blindness worldwide, affecting almost 60 million people in 2020 [1], estimated to increase to 112 million people worldwide by 2040 [2]. Treatment modalities for glaucoma aim to reduce intraocular pressure (IOP), thus halting the progression of the disease. Traditional glaucoma treatments consist of the use of topical antiglaucoma medications, various laser therapies, and penetrating filtration surgeries [3].

Antiglaucoma medications are considered the most common initial treatment for glaucoma [3]. They either suppress aqueous production or increase aqueous outflow. Although effective, many patients demonstrate poor compliance with antiglaucoma medications, which can lead to fluctuations in IOP [4–7]. Furthermore, the cost and side effects associated with antiglaucoma medications, such as ocular surface disease, exacerbate the challenges of patient compliance with the negative consequence of disease progression, leading to vision loss [8–12].

The second most common treatment option is laser therapy, which acts to create a more porous trabecular meshwork structure, thereby increasing aqueous outflow. Among the surgical options, widely used are penetrating filtration surgeries such as trabeculectomy [13] and tube shunt implants, particularly in developing countries. These surgeries involve the formation of a surgical fistula and eventually a bleb which are commonly associated with an increased risk of complications [14]. Moreover, filtration surgeries require a stringent postoperative follow-up to manage both early and late postoperative complications. Trabeculectomy requires wound modulation by the removal of releasable sutures, use of subconjunctival 5-fluorouracil, needling, etc. in the early postoperative period. Valved tube shunts require stringent monitoring during the hypertensive phase, while non-valved tube shunts need manipulation of ligatures [15]. All these require a long recovery period which may not be viable for some patients.

Bridging the gap between medication and traditional surgical options, a number of minimally invasive glaucoma surgeries (MIGSs) have been established, which permit the utilization of less invasive procedures that not only reduce the need for antiglaucoma medications but also help in delaying more invasive surgical procedures like trabeculectomy or shunts [16].

MIGSs aim to either increase trabecular/uveoscleral/subconjunctival outflow or decrease aqueous humour production. Most MIGSs are focal in their approach, inserted at a single site either into the trabecular meshwork or Schlemm's canal, or targeting a single site only within the outflow pathway, but the pathophysiology of glaucoma is known to affect all aspects of the conventional outflow pathway, both proximal and distal. In addition to obstructions within the trabecular meshwork, which have been suggested to account for up to 75% of the total outflow resistance in cases of primary open-angle glaucoma (POAG) [17, 18], herniations of the trabecular meshwork into the collector channels are considered a significant contributor to outflow resistance. Research by Gong and colleagues has shown that up to 90% of the collector channels in POAG eyes are blocked by herniations of the trabecular meshwork [19]. Another important contributor to outflow resistance is Schlemm's canal, which is significantly shorter, narrower, and often collapsed in POAG eyes [20–22].

Canaloplasty offers complete 360-degree catheterization and pressurized viscodilation of Schlemm's canal and is designed to relieve all the points of blockage in the conventional outflow pathway in glaucoma patients. When performed via an ab interno surgical technique, it is a tissue-sparing, stent-free procedure that targets the three main sites of outflow resistance, including the trabecular meshwork, Schlemm's canal, and the collector channels [23–26]. It also avoids conjunctival manipulation and preserves the angle anatomy, thus allowing for future procedures to be performed if required.

Canaloplasty works on the principle of mechanically breaking the adhesions in Schlemm's canal during 360-degree catheterization as well as via viscodilation [23–26]. During viscodilation, a high-molecular-weight hyaluronic acid- (HA-) based ophthalmic viscosurgical device (OVD) is delivered into Schlemm's canal, dilating it and thus opening the distal outflow system. A 250 *μ*m atraumatic bulbous tip enables the iTrack microcatheter to traverse the full circumference of the canal with minimal tissue trauma. The microcatheter is then withdrawn while simultaneously delivering high-molecular-weight OVD via a pressurized mechanism.

This study investigates the clinical effectiveness and safety of canaloplasty performed using the iTrack microcatheter (Nova Eye Medical, Inc., Fremont, California) via an ab interno surgical technique as a standalone procedure and when combined with cataract surgery. This study also evaluates the effectiveness of iTrack canaloplasty in mild to moderate glaucoma vs. severe glaucoma.

## 2. Patients and Methods

### 2.1. Study Design

This is a retrospective, multisurgeon case study wherein we study the effectiveness of canaloplasty performed via an ab interno surgical technique in reducing IOP and medication dependence as a standalone procedure or in combination with cataract surgery in POAG patients, in mild-moderate vs. severe POAG, and controlled (≤18 mmHg) vs. uncontrolled (>18 mmHg) IOP eyes [27, 28]. The eyes that were included in the group successfully controlled with medications at baseline, with an IOP equal to or less than 18 mmHg, were enrolled to reduce patient reliance on medications or due to intolerance to medications.

Baseline ophthalmic examination included history of glaucoma, medication use, IOP, best-corrected distance visual acuity (BCDVA), gonioscopy, slit lamp, fundus examination, and Humphrey's visual field (HVF) test.

Primary endpoints included mean IOP and mean number of antiglaucoma medications at 36 months (percent and number of medications). Secondary outcome measures included comparisons between the standalone and combined procedures, glaucoma severity (mild-moderate vs. severe), and controlled vs. uncontrolled IOP eyes.

All surgeries were performed between January 2017 and November 2018. The study was performed in accordance with the ethical principles of the Declaration of Helsinki, Good Clinical Practice (GCP), and ISO 14155:2011 and applicable local regulations. A procedure consent form was completed for all patients.

### 2.2. Patient Selection

Inclusion criteria involved adult POAG patients (18 years of age or older) with a diagnosis of mild-moderate or severe glaucoma, as per the Hodapp–Parrish–Anderson classification [29], and open-angle structures on gonioscopy, with homogeneously pigmented trabecular meshwork without variegation or excessive pigmentation. Disease severity was determined based on mean deviation (MD) from Humphrey visual field (HVF) testing: mild (0 to −5.99 dB), moderate (from −6 dB to −11.99 dB), and severe cases (−12 dB or worse) [30].

Patients with peripheral anterior synechiae, goniosynechiae, or angle recession were excluded from the study. Patients with neovascular disease, uveitis, peripheral anterior synechiae, and developmental or other forms of secondary glaucoma, such as steroid-induced glaucoma, were also excluded.

### 2.3. Groups

The results are stratified in groups such as follows: whether the procedure was performed in combination with phacoemulsification (combined group) vs. canaloplasty performed as standalone (standalone group); according to baseline disease severity based on MD from HVF testing, eyes were categorized into mild-moderate group vs. severe group; according to baseline IOP, eyes were categorized into controlled group (≤18 mmHg) vs. uncontrolled group (>18 mmHg).

### 2.4. Surgical Technique

The surgical technique was mentioned elsewhere [31]. In brief, trabecular meshwork is punctured with a 25 G needle, and the microcatheter is primed with OVD and then inserted into Schlemm's canal, circumnavigating the entire 360 degrees of the canal. If an obstruction to the passage of the microcatheter is encountered, the microcatheter is withdrawn and a second paracentesis is created to perform the procedure from the opposite direction. After the microcatheter completes 360-degree catheterization, it is slowly withdrawn at a rate of 1 clock hour per second. Simultaneously, precisely regulated microquantity of high-molecular-weightHA-based OVD (Healon GV; sodium hyaluronate: 1.4%) is delivered into Schlemm's canal. In the iTrack + phaco group, cataract surgery was performed prior to canaloplasty.

Postoperative care included a topical steroid like prednisolone acetate 1%, a non-steroidal anti-inflammatory drug (NSAID), and a fourth-generation fluoroquinolone. The antibiotic and NSAID were stopped after 7 days, and the steroid was reduced to twice daily dosing for 2 weeks and then stopped. Antiglaucoma medications were stopped post-op.

### 2.5. Statistical Analysis

The statistical analysis was completed using a commercially available statistical software package (IBM SPSS Statistics for Windows, Version 20.0., IBM Corp., Armonk, NY) and using the statistical software R, release 4.0.3 (R Foundation for Statistical Computing, Vienna, Austria). Data are presented as means and standard deviations for continuous variables and as counts and percentages for categorical variables.

Outcome measures were assessed using descriptive statistics at two time points: baseline and month 36. To compare baseline and post-op time points of the same variable, paired *t* testing was used. To compare post-op means of two different groups, *t*-test for independent samples was used. All *p* values are two-sided and correspond to a significance level of <0.05.

## 3. Results

### 3.1. Patient Demographics

Seventy-two (72) eyes that met the study eligibility criteria and completed the preoperative visit were enrolled in the study. Of the total eyes enrolled, 70% were females (*n* = 37).  Table 1 presents the patient demographics and baseline characteristics. Patients were predominantly Caucasian (81%), with the majority (67%) diagnosed with mild-moderate POAG. Most patients (*n* = 63) underwent a combined procedure (iTrack + phaco), while 9 patients underwent iTrack canaloplasty as a standalone procedure. Of the total 72 eyes, 21 had a history of prior SLT. Microquantity of high-molecular-weightHA-based OVD delivered into Schlemm's was a mean number of 39.4 ± 8.6 clicks (min: 24; max: 60; approximately 2.8 microliters of OVD is delivered per click). Full 360° circumnavigation of Schlemm's canal was achieved in 62 eyes (87%), 320° in 2 eyes, 300° in 3 eyes, and 270° in 4 eyes, with 1 eye not recorded.

### 3.2. IOP and Medication Reduction

Mean IOP preoperatively for the 72 eyes was 18.6 ± 5.8 mmHg, which showed a 25% reduction to 13.5 ± 4.7 mmHg at the last follow-up (*p* < 0.001). Mean number of medications also showed a significant reduction of 29%, decreasing from 2.4 ± 1.0 preoperatively to 1.6 ± 1.2 at the last follow-up (Table 2).

### 3.3. Standalone Procedure vs. Combined with Phacoemulsification

Sixty-three eyes underwent combined ABiC with phacoemulsification, of which 20 eyes (31.7%) had severe glaucoma, and 24 eyes (38.1%) had uncontrolled glaucoma. Mean IOP preoperatively for the combined group (*n* = 63) was 18.5 ± 5.6 mmHg, which showed a 26% reduction to 13.7 ± 4.8 mmHg at the last follow-up (*p* < 0.001). Mean number of medications was also significantly reduced by 32%, from 2.3 ± 1.0 preoperatively to 1.6 ± 1.2 at the last follow-up.

In the standalone group (*n* = 9), IOP showed a 36% reduction from 19.3 ± 7.7 at baseline to 12.4 ± 4.4, while medications reduced from 2.7 ± 0.7 preoperatively to 2.0 ± 1.4 at the last follow-up (Table 2). The IOP reduction was statistically significant (*p*=0.021) while the medication reduction was not (*p*=0.262). The difference between the combined group and standalone group was statistically insignificant for IOP as well as medication reduction (*p*=0.22 and *p*=0.23, respectively). Fifty-six percent (56%) of eyes in the combined group and also 56% of eyes in the standalone group had >20% reduction in IOP.

### 3.4. Mild-Moderate vs. Severe Glaucoma

In the combined group (*n* = 63), mean IOP preoperatively for the mild-moderate eyes (*n* = 43) was 18.8 ± 5.7 mmHg, which showed a 28% significant reduction to 13.6 ± 3.7 mmHg at the last follow-up (*p* < 0.001). Mean number of medications also showed a significant reduction by 40%, reducing from 2.2 ± 1.0 preoperatively to 1.4 ± 1.3 at the last follow-up (*p* < 0.001). One eye (2%) was medication-free at baseline vs. 14 eyes (33%) at the last follow-up.

In the severe cohort (*n* = 20), IOP showed a 23% reduction, from 18.0 ± 5.4 mmHg to 14.0 ± 6.6 mmHg (*p* < 0.004), while medications were 2.5 ± 0.9 preoperatively and 2.1 ± 0.8 at the last follow-up (*p*=0.046) (Table 2). At the last follow-up, 30% of patients in the severe group required ≤1 medication as opposed to 20% at baseline.

### 3.5. Controlled vs. Uncontrolled IOP

In the combined group (*n* = 63), the eyes with preoperative controlled IOP (≤18 mmHg) had a preoperative mean IOP of 15.2 ± 2.2 mmHg which decreased to 12.1 ± 2.6 mmHg at the last follow-up (*p* < 0.001) and the mean number of medications also showed a significant reduction from 2.3 ± 1.0 preoperatively to 1.5 ± 1.1 at the last follow-up (*p* < 0.001).

In the uncontrolled group (>18 mmHg), mean IOP decreased from 24.0 ± 5.1 mmHg to 16.3 ± 6.2 mmHg at the last follow-up (*p* < 0.001) and the mean number of medications reduced from 2.3 ± 1.0 preoperatively to 1.7 ± 1.2 at the last follow-up (*p*=0.001) (Table 2). The reduction in both groups was statistically significant. The difference among the groups at the last follow-up was non-significant (*p*=0.19) for medications and significant for IOP (*p*=0.001).

**Figure 1:**
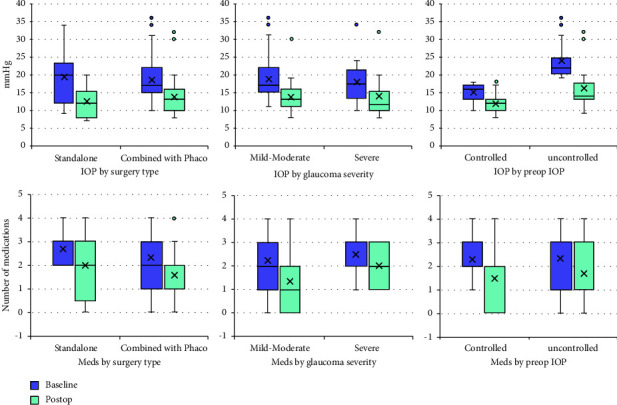
IOP and medications outcomes at baseline and postoperative by surgery type (standalone vs. combined with cataract surgery group), glaucoma severity (mild-moderate vs. severe; only combined with cataract surgery group) and preoperative IOP (controlled vs. uncontrolled; only combined with cataract surgery group).

Table 3 summarizes the preoperative and postoperative mean IOP and number of medications in the combined group, split by number of medications at baseline (0, 1, 2, 3, and 4) in controlled and uncontrolled eyes. The results are shown in Figure 1.

### 3.6. Complications

There was one localized Descemet's membrane detachment in the combined group which resolved spontaneously with no late sequelae.

## 4. Discussion

In this study, canaloplasty performed via an ab interno surgical technique was found to be effective in reducing IOP and medication dependence in all grades of POAG. Specifically, 360-degree catheterization of Schlemm's canal followed by pressurized viscodilation was found to be a good option for sustained reduction by >25% in both mean IOP and mean number of medications, as a standalone procedure as well as in combination with phacoemulsification. This demonstrates the versatility and utility of the procedure in different grades of glaucoma and its compatibility in different glaucoma treatment protocols.

A number of studies indicate the safety and effectiveness of ab interno canaloplasty in lowering IOP and reducing medication in POAG [32, 33]. A 2020 study by Kazerounian et al. reported similar findings, with reduction in mean IOP of 32.5% at 24 months following iTrack canaloplasty. Antiglaucoma medication was also significantly reduced, from 1.92 ± 1.04 at baseline to 0.05 ± 0.23 at 24 months [33]. In 2022, a Gallardo study demonstrated that ABiC reduced IOP from 20.5 ± 5.1 mmHg preoperatively to 13.3 ± 2.1 mmHg (−35%) and the number of medications from 2.8 ± 0.9 preoperatively to 1.3 ± 1.3 at 12–24 and 36 months postoperatively [34]. Khaimi has reported similar results at 36 months with the same number of eyes (*n* = 45 vs. *n* = 44 in Gallardo) [31]. This study demonstrates a similar trend at a mean follow-up time of 3.4 years, with a 25% reduction in mean IOP and 30% reduction in mean number of glaucoma medications. As per the Gallardo and Khaimi studies, the IOP reduction in both the combined and standalone group was statistically significant, whereas in our study, the reduction in medications in the standalone group was not.

In a 2021 study by Gallardo, iTrack canaloplasty was shown to be effective in cases of mild-moderate and severe glaucoma, achieving similar reductions in mean IOP at 24 months at 32.7% and 33.6%, respectively [35]. In the present study, canaloplasty was found to be effective not only in mild-moderate but also severe glaucoma, with 53% patients in mild-moderate group and 60% patients in the severe group demonstrating a >20% reduction in IOP. This is a significant finding as MIGS was initially conceived as a treatment option for reducing IOP and medication dependence in only mild-moderate glaucoma patients. These promising results in severe cases can translate into the utilization of canaloplasty to defer or reduce the need of more invasive filtering surgeries in such cases.

According to the results based on the disease severity, the controlled vs. uncontrolled data also show that the eyes that had a controlled baseline IOP (defined as lower than or equal to 18 mmHg) benefited of a significant reduction in IOP and medications, and those which had a baseline IOP above 18 mmHg were brought to a mean IOP of 16.3 ± 6.2 yet benefiting of a significant reduction in medication of 27%. This is also in line with the literature as recently reported by Koerber and Ondrejka in a 4-year study [36] and suggests that canaloplasty is a viable procedure also to bring under control eyes with a high IOP without compromising on the number of medications required. Table 3 confirms this finding: in controlled eyes, canaloplasty was able to keep IOP under control while reducing the number of medication, except on those eyes which were on only 1 medication baseline. In uncontrolled eyes, canaloplasty was able to decrease IOP while, in some patients, also to reduce the number of medications.

Canaloplasty outcomes are proved in the literature, and this study confirms its efficacy. However, in more advanced glaucoma eyes and those with uncontrolled glaucoma, we have observed that the outcomes have a reduced efficacy in reducing the medication burden, which is probably due to the degree of the preoperative glaucoma stage.

Limitations include the retrospective nature of the study, resulting in potential selection bias. The sample size was relatively small; a larger sample size would permit a more statistically significant finding. The higher proportion of eyes treated with canaloplasty combined with cataract surgery does not allow to separate significantly the effect of cataract surgery on IOP, which is known to contribute to a reduction in IOP (Shingleton et al. who reported an IOP decrease of 1.8 ± 3.5 mmHg at 5 years) [37]. However, the several groups that have been categorized in this study (standalone vs. combined with cataract surgery; mild-moderate vs. severe; controlled vs. uncontrolled) suggest an additional IOP benefit of canaloplasty above that expected for phacoemulsification alone.

## 5. Conclusion

iTrack canaloplasty is emerging as a comprehensive option for glaucoma patients, offering the advantage of being minimally invasive, tissue-sparing, implant-free, and effective as a standalone procedure or in combination with phacoemulsification, in mild-moderate as well as severe glaucoma. It is also found to be useful in patients with a history of previous treatments such as SLT. Thus, it can be a useful addition to the glaucoma treatment algorithm for all POAG patients, reducing the need for antiglaucoma medications and delaying more invasive filtering surgeries.

## Figures and Tables

**Table 1:** Demographics of patients with primary open-angle glaucoma (POAG).

	*Patients (n* *=* *53)*

Gender	
Female	37 (69.8%)
Male	16 (30.2%)

Ethnicity	
African American	2 (3.8%)
Caucasian	43 (81.1%)
Hispanic	4 (7.5%)
Native American	4 (7.5%)

	*Eyes (n* *=* *72)*

Glaucoma severity	
Mild	18 (25.0%)
Moderate	30 (41.7%)
Severe	24 (33.3%)
Eye	
Left	32 (44.4%)
Right	40 (55.6%)

With prior glaucoma surgery	
None	51 (70.8%)
SLT	21 (29.2%)

Follow-up duration (years)	
Mean (SD)	3.43 (0.5)
Median [min, max]	3.36 [2.5, 4.5]

**Table 2:** Mean reduction in IOP and number of medications for (1) all patients; (2) combined group vs. standalone group; (3) mild-moderate group vs. severe glaucoma group; and (4) controlled group vs. uncontrolled group.

	*Baseline (n* *=* *72)*		*Post-op (n* *=* *72)*		*p value*	
IOP						
Mean (SD)	18.6 (5.8)		13.5 (4.7)		<0.001	
Mean reduction	—		24.5%			
Meds						
Mean (SD)	2.36 (1.0)		1.63 (1.2)		<0.001	
Mean reduction	—		29.2%			

	*Combined*			*Standalone*		
	*Baseline (n* *=* *63)*	*Post-op (n* *=* *63)*	*p value*	*Baseline (n* *=* *9)*	*Post-op (n* *=* *9)*	*p value*

IOP						
Mean (SD)	18.52 (5.6)	13.68 (4.8)	<0.001	19.33 (7.7)	12.44 (4.4)	=0.021
Mean reduction	—	26.1%		—	35.6%	
Meds						
Mean (SD)	2.32 (1.0)	1.57 (1.2)	<0.001	2.67 (0.7)	2.00 (1.4)	=0.262
Mean reduction	—	32.2%		—	25.0%	

*Eyes combined with phacoemulsification (N* *=* *63)*						
	*Mild-moderate*			*Severe*		
	*Baseline (n* *=* *43)*	*Post-op (n* *=* *43)*	*p value*	*Baseline (n* *=* *20)*	*Post-op (n* *=* *20)*	*p value*

IOP						
Mean (SD)	18.77 (5.7)	13.56 (3.7)	<0.001	18.00 (5.4)	13.95 (6.6)	=0.004
Mean reduction	—	27.8%		—	22.5%	
Meds						
Mean (SD)	2.23 (1.0)	1.35 (1.3)	<0.001	2.50 (0.9)	2.05 (0.8)	=0.046
Mean reduction	—	39.6%		—	−18.0%	

	*Controlled*			*Uncontrolled*		
	*Baseline (n* *=* *39)*	*Post-op (n* *=* *39)*	*p value*	*Baseline (n* *=* *24)*	*Post-op (n* *=* *24)*	*p value*

IOP						
Mean (SD)	15.15 (2.2)	12.05 (2.6)	<0.001	24.00 (5.1)	16.33 (6.2)	<0.001
Mean reduction	—	20.5%		—	31.9%	
Meds						
Mean (SD)	2.31 (1.0)	1.49 (1.1)	<0.001	2.33 (1.0)	1.71 (1.2)	=0.001
Mean reduction	—	35.6%		—	26.8%	

**Table 3:** Controlled and uncontrolled glaucoma eyes split by medications at baseline (0, 1, 2, 3, and 4 medications).

		Pre-op IOP	Pre-op meds	Post-op IOP	Post-op meds
*Controlled*					
1 med pre-op	Mean	15.8	1.00	12.9	1.00
	SD	2.0	0.0	2.3	0.9
	*n*	9	9	9	9

2 meds pre-op	Mean	15.2	2.00	10.7	1.21
	SD	1.8	0.0	2.1	0.9
	*n*	14	14	14	14

3 meds pre-op	Mean	15.4	3.00	13.0	1.73
	SD	2.8	0.0	2.6	1.1
	*n*	11	11	11	11

4 meds pre-op	Mean	13.4	4.0	12.2	2.60
	SD	1.8	0.0	3.6	1.7
	*n*	5	5	5	5

*Uncontrolled*					
0 meds pre-op	Mean	21.0	0.00	11.0	0.00
	*n*	1	1	1	1

1 med pre-op	Mean	24.0	1.00	14.0	0.50
	SD	5.2	0.00	2.6	0.55
	*n*	6	6	6	6

2 meds pre-op	Mean	21.0	2.00	11.0	1.00
	SD	1.4	0.00	2.8	0.0
	*n*	2	2	2	2

3 meds pre-op	Mean	24.8	3.00	18.6	2.36
	SD	5.7	0.00	7.0	1.0
	*n*	14	14	14	14

4 meds pre-op	Mean	22.0	4.00	14.0	3.00
	*n*	1	1	1	1

## Data Availability

All data generated or analysed during this study are included in this article. Further enquiries can be directed to the corresponding author.
